# Endothelial cell pseudopods and angiogenesis of breast cancer tumors

**DOI:** 10.1186/1475-2867-5-17

**Published:** 2005-05-26

**Authors:** Ivan L Cameron, Nicholas Short, LuZhe Sun, W Elaine Hardman

**Affiliations:** 1 Department of Cellular and Structural Biology, University of Texas Health Science Center at San Antonio, 7703 Floyd Curl Drive, San Antonio, TX 78229, USA; 2 Pennington Biomedical Research Center, Louisiana State University, 6400 Perkins Road, Baton Rouge, LA 70808, USA

**Keywords:** endothelial cell sprouting, breast cancer, angiogenesis

## Abstract

**Background:**

A neoplastic tumor cannot grow beyond a millimeter or so in diameter without recruitment of endothelial cells and new blood vessels to supply nutrition and oxygen for tumor cell survival. This study was designed to investigate formation of new blood vessels within a human growing breast cancer tumor model (MDA MB231 in mammary fat pad of nude female mouse). Once the tumor grew to 35 mm^3^, it developed a well-vascularized capsule. Histological sections of tumors greater than 35 mm^3 ^were stained with PAS, with CD-31 antibody (an endothelial cell maker), or with hypoxia inducible factor 1α antibody (HIF). The extent of blood vessel and endothelial cell pseudopod volume density was measured by ocular grid intercept counting in the PAS stained slides.

**Results:**

The tumor area within 100–150 μm of the well-vascularized capsule had few blood vessels and only occasional endothelial cell pseudopods, whereas the area greater than 150 μm from the capsule had more blood vessels, capillaries, and a three-fold increase in volume density of pseudopods sprouting from the capillary endothelial cells. This subcortical region, rich in pseudopods, some of which were observed to have vacuoles/lumens, was strongly positive for presence of HIF. In some larger tumors, pseudopods were observed to insinuate for mm distances through hypoxic regions of the tumor.

**Conclusion:**

The positive correlation between presence of HIF and the increased extent of pseudopods suggests volume density measure of the latter as a quantifiable marker of tumor hypoxia. Apparently, hypoxic regions of the tumor produce HIF leading to production of vascular endothelial growth factors that stimulate sprouting of capillary endothelial cells and formation of endothelial cell pseudopods.

## Background

Most tissues in the non-growing adult body have an adequate vascular supply with no need for formation of new blood vessels. Growth in tissue or tumor mass however requires recruitment of endothelial cells to form new blood vessels. In an early report, new blood capillaries were directly observed to form by sprouting of pseudopods from existing endothelial cells [1]. Speidel reported endothelial cell sprouting in the regenerating tail fin of the frog (*Rana clamitans*) 6 day after amputation. He saw capillary sprouts that progressed for 32 μm through the tissue over a 50-minute period, leaving a solid capillary sprout behind. Up to seven side sprouts or pseudopods also arose along the length of this main capillary sprout during this 50-minute period. He also observed a sprout that acquired a vacuole that fused with the capillary lumen and allowed entry of an erythrocyte from the capillary lumen. This report of morphological events involved in capillary cell sprouting and pseudopod formation remains the most definite description of endothelial cell sprouting and pseudopod formation.

The recruitment of endothelial cells by a tumor is a key early step in tumor angiogenesis [2]. This tumor angiogenic process has become an important target in cancer therapy [2-4]. There is now strong molecular and genetic support for targeting tumor angiogenesis [2-4]. However, there is much more to be learned about tumor angiogenesis. In our own investigation of angiogenesis of the human breast cancer tumors (MDA MB231) growing in the female nude mouse xenograft, we discovered that staining 8 μm thick histological mid-cross sections of the tumor with the periodic acid-Schiff (PAS) technique for glycoproteins [5] revealed an unexpected abundance of PAS positive endothelial cell pseudopods. The distribution of endothelial cell pseudopods was observed not to be random throughout the tumor. This is the report of a study done to determine the spatial pattern and the possible explanation for this non-random pattern of endothelial cell pseudopods during tumor angiogenesis.

## Results

**Figure 1:**
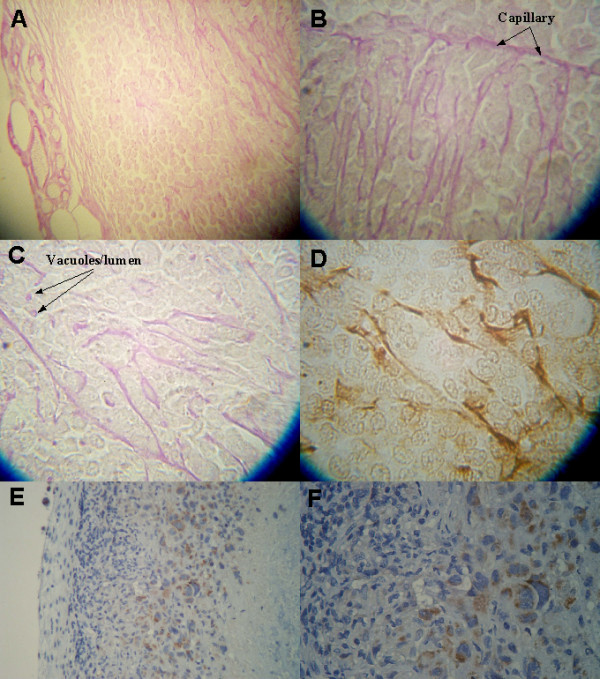
Photomicrographs illustrating the pattern of the tumor vascularization. A, The tumor capsule (left) of this PAS stained section reveals blood vessels. The cortex under the capsule reveals no blood vessels and few endothelial pseudopods while the subcortical area to the top right has more pseudopods. B, at higher magnification the subcortical area of the tumor reveals a blood capillary with multiple endothelial pseudopods protruding at right angles into the tumor mass. C, Endothelial pseudopods are seen to branch and occasionally have a vacuole/lumen (arrow). D, The endothelial pseudopods react immunohistochemically positive for the CD-31 specific endothelial cell marker, using the avidin-biotin peroxidase complex method. E, Viable cell area can be seen beneath tumor capsule (left) while necrotic area can be seen to the right. F, Enlarged subcortical area from E. In E, the HIF-α positive area between the viable and the necrotic tissue is stained brown.

A total of 10 tumors greater than 35 mm^3 ^were fixed, embedded in paraffin wax and midsections were cut at 8 μm thickness for staining and histological study. Histological examination of midsections of PAS stained tumors was done to assess the tumor vascularization patterns. Tumors with a volume >35 mm^3 ^demonstrated a connective tissue capsule with blood vessels (Fig. 1A). At higher magnification the cortical area within about 100 μm of the capsule revealed few blood vessels while the area greater than 100 μm from the capsule showed considerable evidence of blood vessels and of capillaries with many endothelial pseudopods extending away from the capillaries (Fig. 1B&C). The general direction of the pseudopods was parallel to the tumor capsule surface. Immunohistochemical localization of CD-31, used as a specific marker of blood vessel endothelium, demonstrated a positive reaction of pseudopods (Fig. 1D). Areas of tumor necrosis were observed below the subcortical area (Fig. 1E&F). Immunohistochemical localization of hypoxia inducible factor 1-α (HIF) reveals the subcortical area of the tumor to contain HIF positive cells while the tumor capsule, cortex and necrotic areas of the tumor demonstrate no evidence of HIF. Thus, the areas found to be HIF positive were enriched in endothelial pseudopods.

**Figure 2:**
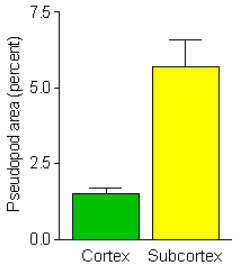
Quantification of volume density differences of endothelial cell pseudopods in the tumor cortex, within 100 to 150 μm of the well-vascularized tumor capsule, and in the subcortical region at a distance of greater than 150 μm beneath the tumor capsule. The mean ± SEM of the pseudopod volume density of 10 tumors in the two different regions of the tumors indicate a significant (p < 0.001) three fold higher value of pseudopod volume density in the subcortical region compared to the cortical region of the tumor.

Ocular grid intercept counting was used to quantify tumor vascularization in 8 μm thick PAS stained histological sections of the tumors. The numbers of ocular grid intercepts were scored over: 1.) blood vessels and capillaries, 2.) endothelial pseudopods and 3.) areas with no indication of these structures. This method has been shown to be a usable measure of volume density occupied by recognizable structures. The results of the scoring of blood vessels and of endothelial pseudopods in the subcortical regions of the tumors are summarized in Fig. 2. As illustrated in Fig. 2, there was significantly less pseudopod volume density in the cortical versus the subcortical region of the tumor.

Can the observation of PAS positive endothelial cell pseudopods be generalized to human breast cancer surgical biopsy specimens? To address this question, specimens from 15 breast cancer patients, obtained from the University of Texas Health Science Center at San Antonio Department of Pathology, were examined. Of these 15 patients, nine were diagnosed with ductal confinement intraductal carcinoma, or "DCIC." Although five showed multilayering of cancer cells within the ducts, none showed evidence of endothelial pseudopods within this cancer cell population. Seven of the biopsy specimens were diagnosed as invasive ductal carcinoma, or "IDS." Six of the seven IDS specimens showed evidence of endothelial pseudopods but to a lesser extent than in the MDA MB231 human breast cancer tumor used in the current study. Two IDS tumor specimens had visible regions of necrosis. The region of the tumor adjacent to these necrosis regions had more pseudopods than seen elsewhere in the tumor. Thus, PAS positive endothelial cell pseudopods were commonly present in the human IDS specimens.

## Discussion

**Figure 3:**
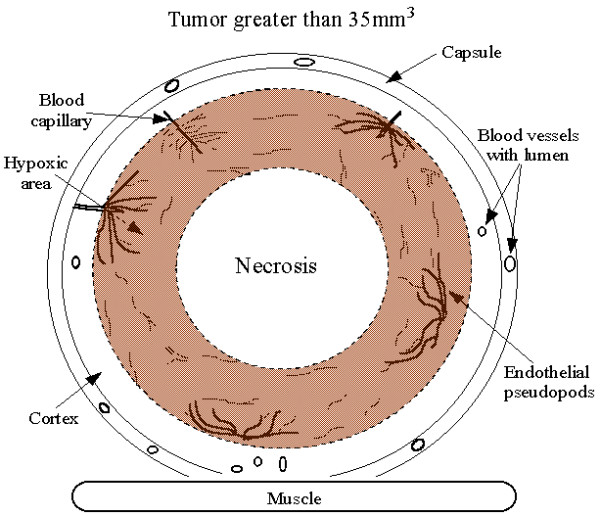
Model of vascularization as observed in the human MDA MB231 breast cancer cell tumor grown as a xenograph in a female nude mouse. The well vascularized tumor capsule overlies the tumor cortex. The cortex extends between 100 and 150 μm beneath the capsule. The HIF-α positive region is located in the subcortical region, at distances greater than 150 μm of the capsule. More endothelial pseudopods are located in this subcortical region than are located in the cortical region of the tumor. Even further away from the vascularized capsule is the region of tumor necrosis which contains no blood vessels or endothelial cell pseudopods. This model is consistent with the conclusion that the hypoxic regions of the tumor produce HIF leading to production of vascular endothelial growth factors and sprouting of endothelial cell pseudopods.

The PAS stained histological sections of the tumors were useful for identification of the spatial pattern of tumor vascularization. Not only could blood vessels and capillaries be identified in the tumor but numerous pseudopods were also observed sprouting from capillaries. The positive reaction to the CD-31 endothelial marker confirmed that the PAS stained structures were blood vessels, capillaries and pseudopods of endothelial cell origin. Figure 3 is a drawing that summarizes the observed distribution of blood vessels and endothelial cell pseudopods. Tumors greater then 35 mm^3 ^revealed distinct areas of viable and necrotic tissue. An area of viable tumor cells was observed beneath the vascularized tumor capsule. The area of viable tumor cells had two distinct subdivisions. The cortical areas of the tumor within 100 to 150 microns of the tumor capsule was not well vascularized and had few endothelial pseudopods whereas the subcortical area further than 100 to 150 microns from the tumor capsule had blood vessels and had many more endothelial pseudopods. Even further beneath the tumor capsule, in the areas of tumor necrosis, there was no evidence of blood vessels, capillaries or endothelial pseudopods.

The observed localization of HIF in the tumors by immunohistochemistry helped explain the vascular pattern of the encapsulated tumors. The HIF positive reactivity in the tumor was localized in the subcortical areas of the tumor in the same area found to have the most endothelial cell pseudopods (Fig. 2). These findings suggest that this subcortical area of the tumor is hypoxic and is producing angiogenesis growth factors, which in turn acts on the host endothelial cells to sprout pseudopods. Sprouting of cultured endothelial cells in response to vascular endothelial growth factors has been reported [6]. In the same report, Feraud et al. demonstrated that several angiostatic agents could inhibit this endothelial cell sprouting. The presence of pseudopodial like structures in viable areas of a tumor immediately adjacent to necrotic areas of the tumor has been previously reported [7]. The lack of HIF reactivity beneath the well vascularized tumor capsule in the cortical area suggests this cortical area is not hypoxic and not in need of an extensive vasculature. Apparently, the PAS staining of endothelial cell pseudopods in tumors gives quantifiable spatial information on where in the tumor that HIF and vascular endothelial growth factors are located.

## Materials and Methods

A detailed account of the materials and methods used in this study has been published elsewhere [8]. Thus, only a summary is given here. Fifteen 6-week-old female athymic nude mice where fed AIN-76 semipurified diet altered to contain 10% corn oil. Two millions human breast cancer cells were inoculated into the inguinal mammary fat pad of each mouse. Once the tumors had grown to greater than 35 mm^3 ^the mice were euthanized by injection of a ketamine/rompun anesthesia supplied by the University of Texas Health Science Center at San Antonio Laboratory Animal veterinarian, then cervically dislocated and exsanguinated by cardiac puncture. The tumors were then removed and fixed in Omni Fix II (Mt. Vernon, NY), dehydrated and embedded in paraffin, sectioned at 4 or 8 μm thickness, mounted on slides, deparaffinized, and stained with H&E or with periodic acid-Schiff (PAS). Additional slides were stained for the immunohistochemical markers for endothelial cells, CD-31 (PECAM-1, PharMingen) or for hypoxia-inducible factor 1-alpha (#OSA-601, Stressgen). Quantification of the volume density of endothelial cell pseudopods was done on the 8 μm thick PAS stained histological sections of the tumors using counts of the number of ocular grid line intercepts.

Archived surgical biopsy specimens that had been fixed in 10% neutralized formalin were dehydrated and embedded in paraffin. Histological sections of the specimens were stained with H&E for routine histopathology examination or with PAS to assess endothelial cell pseudopod distribution.
